# Impact of the Programa Mais médicos (more doctors Programme) on primary care doctor supply and amenable mortality: quasi-experimental study of 5565 Brazilian municipalities

**DOI:** 10.1186/s12913-020-05716-2

**Published:** 2020-09-15

**Authors:** Thomas Hone, Timothy Powell-Jackson, Leonor Maria Pacheco Santos, Ricardo de Sousa Soares, Felipe Proenço de Oliveira, Mauro Niskier Sanchez, Matthew Harris, Felipe de Oliveira de Souza Santos, Christopher Millett

**Affiliations:** 1 Public Health Policy Evaluation Unit, Imperial College London, London, UK; 2 Department of Global Health and Development, London School of Hygiene & Tropical Medicine, London, UK; 3 Departamento de Saúde Coletiva, Universidade de Brasília, Brasília, DF Brazil; 4 Departamento de Promoção da Saúde, Universidade Federal da Paraíba, João Pessoa, PB Brazil; 5 Department of Primary Care and Public Health, Imperial College London, London, UK; 6 Secretaria Estadual de Saude Paraíba, João Pessoa, PB Brazil

**Keywords:** Brazil, Human resources for health, Mortality, Primary care, Doctors

## Abstract

**Background:**

Investing in human resources for health (HRH) is vital for achieving universal health care and the Sustainable Development Goals. The *Programa Mais Médicos* (PMM) (More Doctors Programme) provided 17,000 doctors, predominantly from Cuba, to work in Brazilian primary care. This study assesses whether PMM doctor allocation to municipalities was consistent with programme criteria and associated impacts on amenable mortality.

**Methods:**

Difference-in-differences regression analysis, exploiting variation in PMM introduction across 5565 municipalities over the period 2008–2017, was employed to examine programme impacts on doctor density and mortality amenable to healthcare. Heterogeneity in effects was explored with respect to doctor allocation criteria and municipal doctor density prior to PMM introduction.

**Results:**

After starting in 2013, PMM was associated with an increase in PMM-contracted primary care doctors of 15.1 per 100,000 population. However, largescale substitution of existing primary care doctors resulting in a net increase of only 5.7 per 100,000. Increases in both PMM and total primary care doctors were lower in priority municipalities due to lower allocation of PMM doctors and greater substitution effects. The PMM led to amenable mortality reductions of − 1.06 per 100,000 (95%CI: − 1.78 to − 0.34) annually – with greater benefits in municipalities prioritised for doctor allocation and where doctor density was low before programme implementation.

**Conclusions:**

PMM potential health benefits were undermined due to widespread allocation of doctors to non-priority areas and local substitution effects. Policies seeking to strengthen HRH should develop and implement needs-based criteria for resource allocation.

## Background

Investments in Human Resources for Health (HRH) which deliver sufficient numbers of highly-skilled, motivated and equitably distributed health professionals [1] are essential to achieve universal health care and health targets in the Sustainable Development Goals (SDGs). However, chronic HRH shortages persist in many settings with an estimated 10.3 million extra health workers needed globally to achieve UHC [2]. Interventions to address HRH deficiencies are essential, but so is ensuring implementation as planned to achieve their intended benefits.

Brazil, like many other low- and middle-income countries (LMICs), suffers substantial HRH challenges. Supply of doctors is low by international standards (180 per 100,000 population in 2013 [3]) with large geographic distributional inequalities. Many doctors work in the private hospital sector in urban areas rather than the public system where working conditions and career prospects are perceived to be poor [4, 5]. In 2012, over 42% of the population lived in areas with fewer than 25 doctors per 100,000 [6] – comparable to doctor densities in countries of sub-Saharan Africa (20 per 100,000) [7]. HRH shortages in primary care are especially problematic and contribute to suboptimal health outcomes and continued health inequalities in the country [8]. Brazil’s *Estratégia Saúde da Família* (ESF) is a globally-renowned public primary care system that has delivered improvements in health outcomes [9–13]. However, expansion of the ESF in urban or remote areas has stalled in recent years due to HRH shortages [14].

In 2013, the Brazil government initiated the *Programa Mais Médicos* (PMM) (More Doctors Programme) to expand the number of doctors in under-served areas. In addition to funds for clinic construction and refurbishment, and new medical schools in areas lacking doctors, there was a politically-contentious “emergency expansion” of primary care doctors. These were predominantly Cuban doctors who entered Brazil following an agreement between Cuba and Brazil, organised by the Pan American Health Organisation (PAHO). One year later, in July 2014, there were 14,462 PMM doctors: 79.0% Cubans (11,429), 15.9% Brazilians (2302) and 5.1% other foreigners (731) [15]. By Sept 2015, the total had increased to 17,625 doctors [3]. Studies show that following the PMM there are now fewer municipalities with primary care doctor shortages [16, 17], access to doctors has increased, users are more satisfied, and services have improved including patient-doctor relations, continuity, and coordination [18, 19]. There is also evidence of reductions in hospitalisations, but no effect on infant mortality [20, 21]. However, little is known about PMM’s impact on existing primary care services or adult mortality. Primary care expansion in Brazil led to reductions in child and amenable adult mortality [10], health inequalities [11], and ambulatory care sensitive hospitalisations [9], and therefore health improvements from PMM are anticipated.

Criteria for allocating PMM doctors to Brazil’s 5565 municipalities were developed prior to programme implementation, and based on indicators of local deprivation rather than a formal health needs assessment [22]. Actual allocation was widespread with 4509 (81.0%) municipalities receiving PMM doctors, including municipalities already achieving international benchmarks for doctor supply. PMM implementation provides important policy learning opportunity about prioritising scarce HRH to maximise health system performance and health outcomes [23]. This study examined the impact of PMM on primary care doctor supply and amenable mortality using longitudinal data from 5565 Brazilian municipalities between 2008 and 2017. It explored whether these relationships differ in areas prioritised under PMM allocation criteria, compared with those that were not, and by primary care doctor density prior to PMM introduction.

### Key components and policies changes of the *Programa Mais Médicos* (PMM)

The Brazilian government launched *Programa Mais Médicos* (PMM) in July 2013 with three main components – an “emergency” expansion of primary care doctors, establishing new medical schools with increases in primary care residency positions, and funds for clinic construction and refurbishment.

The “emergency” expansion was the most visible and politically contentious component of PMM. Initially, local managers requested 16,000 primary care doctors to fill vacancies in underserved areas. Competitive salaries were offered under PMM to encourage Brazilian doctors to relocate, but only 1096 Brazilian doctors enrolled [15]. An international cooperation agreement between Brazil and Cuba facilitated by PAHO provided Cuban doctors to fill these vacancies following basic training in primary care and supervision from an accredited university [3, 24]. Municipalities were prioritized to receive PMM doctors using federally-set criteria: municipalities with 20% or more of the population in extreme poverty; 100 municipalities with more than 80,000 population and the lowest income per capita; state capitals, metropolitan regions, and other municipalities encompassing areas with extreme poverty; and municipalities with low/very low human development index or considered vulnerable (e.g. semi-arid or *Quilombo* communities) [6]. However many non-priority municipalities received PMM doctors [6] as the Brazilian Ministry of Health did not adhere to these criteria.

The costs of the PMM were substantial. In 2014, medical provision costs were US$ 1.1 billion (R$ 2.36 billion), 93% financed by the federal budget, or approximately US$ 6000 (R$ 14,000) per month per doctor [25]. Funding for clinic construction and refurbishment exceeded R$5 billion [3]. By 2019, 13,000 new undergraduate medical places had also been created. However, construction and refurbishment of clinics and establishment of new medical schools was paused following a federal funding freeze.

In November 2018, the Cuban government withdrew all Cuban doctors from Brazil citing critical comments by President-elect Jair Bolsonaro. During the election period, he questioned the quality of their training and described them as “slaves” due to the low pay they receive (relative to payments made to the Cuban government) [5]. In August 2019 the government launched a new program called *“Medicos Pelo Brasil”* (Doctors for Brazil), but as of November 2019, approximately 5000 positions remain vacant.

## Methods

### Study design

This study employed differences in differences approaches using longitudinal (panel) regression models to compare the supply of primary care doctors and mortality amenable to healthcare before and after PMM introduction between municipalities that received the programme and municipalities that did not. It uses a panel dataset of 5565 Brazilian municipalities over the period 2008–2017. Longitudinal (panel) regression models are widely employed for programme evaluations [26] and exploit the varied roll out of the PMM programme over time across Brazilian municipalities.

### Data sources and variables

Multiple publicly available data sources were collated. Official statistics on mortality, doctors, hospital beds, private health insurance plans, and municipal health expenditures were collated from the Brazilian Ministry of Health website. Data on PMM implementation was obtained from the Brazilian Ministry of Health including the number and nationality of PMM doctors in each municipality. The website of the Brazilian National Institute of Geography and Statistics (*Instituto Brasileiro de Geografia e Estatistica* - IBGE) was consulted for municipal-level data on population, gross domestic product (GDP), Bolsa Familia expenditure, and sociodemographic characteristics.

The main outcome variables of interest were public primary care doctor density per 100,000 (defined as doctors working in public primary care) and mortality amenable to healthcare (per 100,000 population) – both expressed at the municipal level. Quarterly data on doctor numbers in a municipality and their employment hours were used to generate full time equivalents (FTEs) based on a 40-h working week. Primary care doctors were identified as those reporting ambulatory working hours in primary care facilities (health centres and posts, family health units, mixed healthcare units, water-based clinics serving fluvial communities, and indigenous health centres). Public primary care doctor density was further subdivided into PMM and non-PMM doctors - identified by their contract with the Ministry of Health. A variable denoting the percentage of PMM doctors that were Brazilian was generated for each quarter-municipality observation.

Amenable mortality rates were generated from official death statistics which were encoded based on reported ICD10 codes and age [27] – an approach employed previously [10]. Additionally, groups of amenable deaths were encoded based on categories of causes (see Additional file 1). Annual municipal population estimates were interpolated to generate quarterly observations and, using the age distribution of the population for each municipality from the latest census, denominator populations under 75 years of age were estimated.

A binary variable indicating the presence of PMM in a municipality was our exposure variable. It was defined as any PMM doctor operating in a municipality for each quarter-year to account for the time-varying nature of implementation in each municipality and that PMM doctors could leave municipalities.

### Statistical analyses

Differences-in-differences analyses were employed with multiple time points using longitudinal fixed effects regression models. These models were used to identify associations between PMM introduction and changes in outcomes over time by comparing changes in mortality rates between areas that received the PMM and those that did not before and after PMM implementation. Existing studies examining hospitalisations and healthcare utilisation have employed similar analytical strategies and demonstrate the appropriateness of these approaches and validity of underlying assumptions [20, 21, 28, 29].

The regression models adjust for municipality fixed effects (rather than random effects specification) to control for time-invariant differences between municipalities. They further adjusted for state-quarter-year fixed effects and time-varying municipality characteristics. Time-varying confounders were chosen as proxies of socioeconomic and demographic characteristics of municipalities and to capture wider changes in the health system as used in similar studies [10, 11, 30–32]. Specifically there were: municipal health expenditure (R$) per capita; private insurance plan coverage (%); hospital bed density (beds per 1000 population); municipal Gross Domestic Production (GDP) per capita (R$); Bolsa Familia expenditure per poor person (R$), municipal illiteracy rate (%) (of those aged 15 years or more); percentage households with inadequate sanitation (%); municipal urbanisation rate (%); average municipal income per capita (R$); and percentage households with no electricity (%).

Firstly, trends were explored descriptively and the effect of PMM implementation on doctor density and amenable mortality modelled using longitudinal fixed effects regression models. Models exploring mortality were weighted by average municipal population over the period to provide estimates relevant to an average individual (rather than an average municipality). Robust standard errors (clustered by municipality) were used to account for heteroskedasticity and autocorrelation [33]. Data was analysed using quarterly observations, but effect sizes for mortality were reported as annual to aid interpretability.

Secondly, heterogeneity in PMM impact was assessed by allocation criteria and baseline primary care doctor density. Municipalities were grouped into either priority or non-priority based on allocation criteria. A priority municipality was one that met any of the criteria. Municipalities were also divided into five equal sized groups (quintiles) based on the mean density of primary care doctors (FTE primary care doctors per 100,000 population) in the period prior to PMM implementation (2008–2012). Interactions with the indicator of PMM introduction were used to explore subgroup differences in separate models for programmatic priority and baseline doctor density quintiles. To aid interpretation, the reported effect sizes are calculated as the effect of PMM introduction in each group or quintile (rather than marginal effect sizes relative to Q1 as commonly reported).

Thirdly, heterogeneity between Brazilian PMM and foreign PMM doctors was assessed to explore whether differential PMM impacts existed by the percentage of PMM doctors that were foreign. A categorical variable was created denoting the combination of PMM implementation and percentage of PMM doctors that were Brazilian. Specifically: no PMM implementation; < 20% PMM doctors Brazilian; 20–80% PMM doctors Brazilian; and > 80% PMM doctors Brazilian. This variable was used in the main regression model described above.

### Sensitivity analyses

Model specifications with variations in time and fixed effects were tested. These included additional state-quarter-year fixed effects and state-year-quarter linear time trends. Univariate analyses and stepwise addition of covariates were undertaken to explore stability of effect estimates and the models. In addition to step changes following PMM introduction, slope changes (in underlying trends) were also tested. The PROVAB programme (Program to Value Primary Healthcare Professionals; *Programa de Valorização do Profissional da Atenção Básica*) was operational at a similar time to the PMM. It offered training for primary care doctors which was incorporated into the PMM in 2016 (allowing these doctors to also enter the PMM). Possible bias from co-introduction of this programme was tested by repeating regression analyses with PROVAB-PMM doctors omitted. Models with alternative covariate specifications, including linear time trends interacted with baseline covariate values (2008 Q1) were used to assess the robustness of the findings. Additionally, inverse probability weighting of treatment (IPTW) was employed to test whether further weighting of municipalities by baseline covariates would affect regression results. An event study analysis was also carried out on doctor density outcomes and amenable mortality to examine evidence of pre-trends and test robustness of the findings to alternative analytical approaches.

## Results

**Fig. 1:**
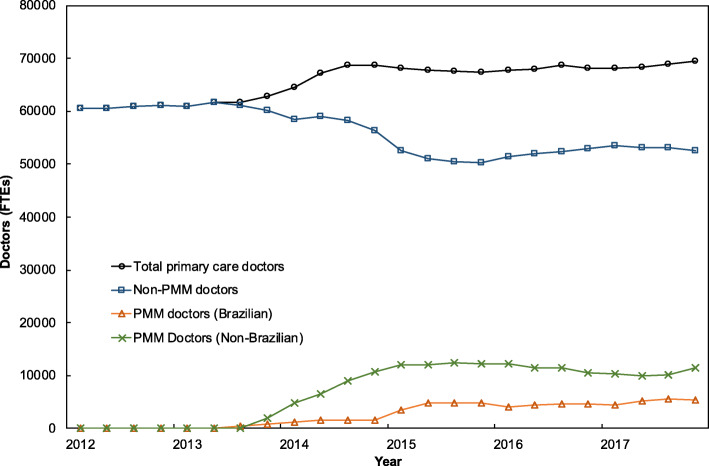
Total number of primary care doctors working in public system in Brazil 2012–2017. Source: CNES, Ministry of Health and author’s own work

Total FTE doctors in Brazil increased from 268,970 in Q1 2008 to 418,566 in Q4 2017 representing an increase from 145.1 to 210.6 per 100,000 population (see Additional file 1). However, the proportion working in the public sector decreased (from 86.8 to 75.3%). Total full-time equivalent (FTE) doctors working in primary care in the public system (hereafter ‘primary care doctors’) grew 14.2% from 60,900 to 69,536. Although, as the number of doctors in other parts of the public system increased, the proportion of primary care doctors declined (from 26.1 to 22.1%) (Fig. 1).

Over the period 2013–2017, 35,060 PMM doctors were contracted - 72.8% of these were non-Brazilian. The peak number of PMM doctors working was in Q1 2016 with 18,088 doctors in operation. Of 5565 municipalities, 4509 (81.0%) ever received a PMM doctor between 2013 and 2017, but this averaged 4000 municipalities from mid-2015 onwards (see Additional file 1). This compares to the 46.5% (2589) of municipalities prioritised for PMM allocation under programme criteria; including 1527 due to high levels of extreme poverty, 486 capitals or in metropolitan areas, 98 classified as populations over 80,000 and low incomes, and 478 under other vulnerabilities (semi-arid, low human development index, indigenous areas).

**Fig. 2:**
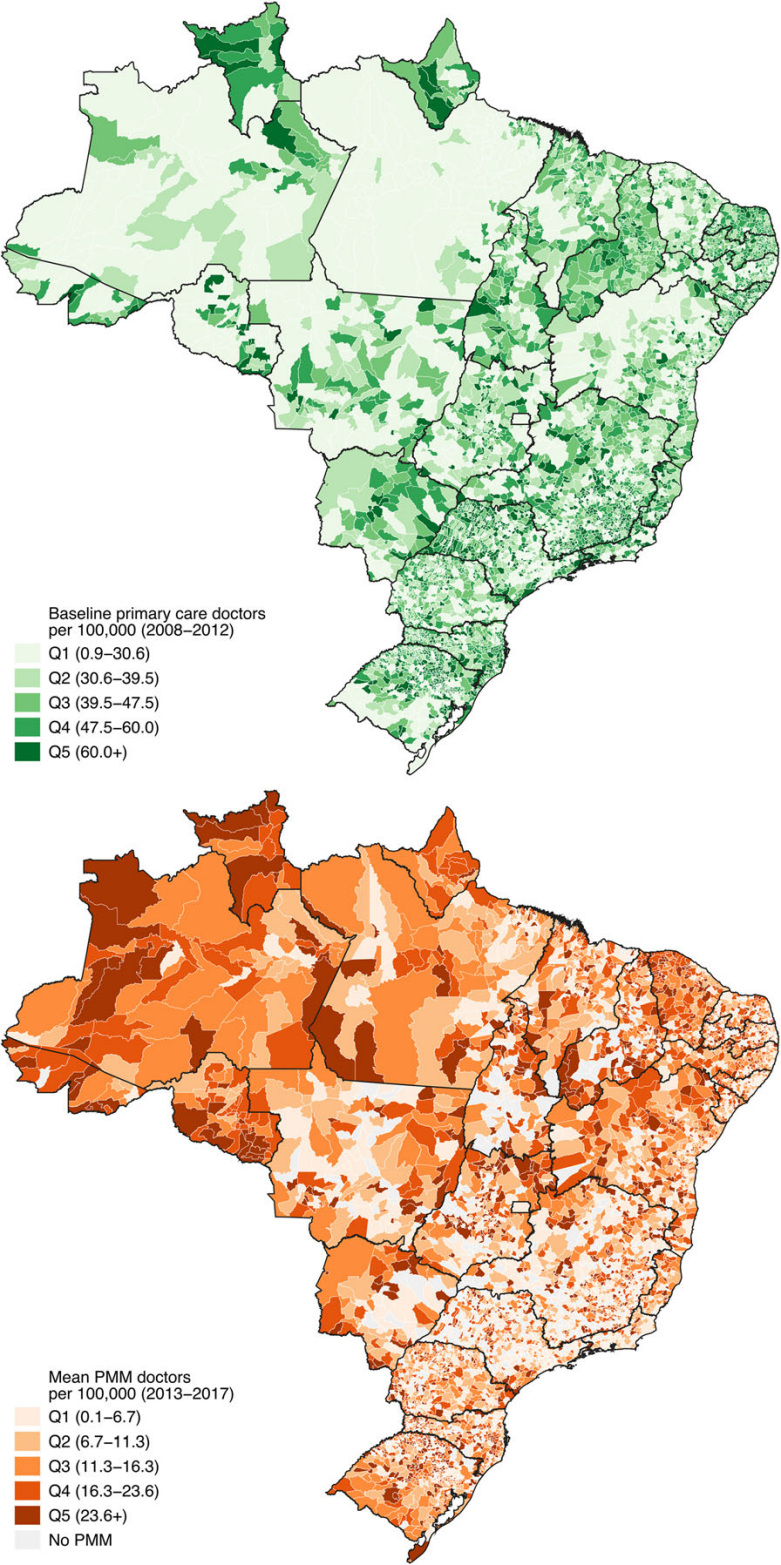
Baseline primary care doctor density and mean PMM doctor density across Brazilian municipalities. Source: CNES, Ministry of Health and author’s own work

PMM introduction coincided with increases in total primary care doctors, but there were concurrent reductions in the number of non-PMM primary care doctors. The mean number of municipal primary care doctors (FTEs) per 100,000 grew 11.0% from 46.1 in Q1 2012 to 51.6 in Q4 2017. Regions with lowest primary care density at baseline were mainly in the north and north east of Brazil and were also the areas that had the largest increases in doctor density following PMM introduction (Fig. 2). In adjusted differences-in-differences models, PMM introduction was associated with an increase of 5.7 (95%CI, 5.1, 6.4) total primary care doctors per 100,000 (Table 1) representing a 12.2% increase on a baseline of 47.2 doctors per 100,000. This overall change was made up of a 15.1 increase (95%CI, 14.9, 15.5) in PMM doctors per 100,000 population, but also a reduction of − 9.4 (95%CI, − 10.0,-8.8) per 100,000 in non-PMM doctors.

**Table 1:** Effect of PMM on primary care doctor density and mortality amenable to healthcare

	Total primary care doctor density	95%CI	PMM Doctor density	95%CI	Non- PMM Doctor density	95%CI	Amenable mortality rate	95%CI
PMM Introduction	5.74***	5.11,6.37	15.14***	14.80,15.48	−9.40***	−10.03,-8.77	−1.06**	− 1.78,-0.33
Health expenditure (R$) per capita	13.17***	10.20,16.14	4.87***	3.68,6.06	8.30***	5.66,10.94	2.95*	0.17,5.86
Private insurance plan coverage (%)	0.02	−0.06,0.11	0.02	− 0.00,0.05	0.00	− 0.08,0.08	0.34***	0.26,0.41
Hospital beds per 1000 pop	0.00	−0.36,0.36	0.07	− 0.06,0.20	− 0.07	− 0.43,0.28	0.81***	0.40,1.21
GDP per capita	−17.90	−44.63,8.83	− 15.17*	−27.39,-2.95	−2.73	−29.11,23.64	−7.81	−34.68,19.06
Bolsa Familia expenditure per poor person	0.01***	0.01,0.02	0.02***	0.01,0.02	−0.00	− 0.01,0.00	0.02***	0.02,0.03
Illiteracy rate (15 + year)	−0.17	−0.43,0.09	− 0.04	−0.19,0.11	− 0.13	−0.39,0.13	0.63***	0.28,0.97
Households with inadequate sanitation (%)	−0.11***	−0.18,-0.05	0.04	−0.00,0.08	− 0.15***	−0.22,-0.08	− 0.14*	−0.25,-0.02
Urbanisation rate (%)	0.03	−0.05,0.10	0.09***	0.06,0.13	−0.06	−0.14,0.01	0.10*	0.02,0.29
Income (R$) per capita	−0.00	−0.01,0.01	− 0.00	−0.01,0.00	− 0.00	−0.01,0.01	− 0.02***	−0.03,-0.01
Households with no electricity (%)	−0.29***	−0.38,-0.20	− 0.10***	−0.15,-0.05	− 0.19***	−0.29,-0.09	0.29***	0.12,0.46
*N* (municipalities)	5565		5565		5565		5565	
*N* (observations)	222,600		222,600		222,600		222,600	

* *p* < 0.05, ** *p* < 0.01, *** *p* < 0.001; Cluster robust standard errors employed; Doctor densities refer to full-time equivalents per 100,000 population; Amenable mortality rate is expressed per 100,000 population and reported as annual effect sizes; Amenable mortality regression results are weighted by municipal population

Over the period 2008–2017, there were 7,431,535 deaths of individuals aged under 75 years, with 21.5% of these (1,594,117) amenable to healthcare. The weighted-mean annual municipal amenable mortality rate increased from 81.1 deaths per 100,000 in 2008 to 85.7 in 2017. Adjusted difference-in-differences analysis showed that PMM introduction was associated with an immediate (step-change) lower amenable mortality rate of − 1.06 deaths per 100,000 (95%CI, − 1.78 to − 0.34) per year (Table 1). There was no indication of ongoing reductions (slope change) in amenable mortality following PMM introduction over above annual trends (see Additional file 1). With an average annual amenable mortality rate of 83.6 deaths per 100,000 over the period, this corresponds to a 1.3% reduction. This effect was predominantly driven by reductions in respiratory diseases including influenza and pneumonia (see Additional file 1).

Exploring heterogeneity of impact by municipality prioritisation for PMM (Table 2) revealed larger increases in primary care doctor density following PMM introduction in non-priority (7.43 doctors per 100,000 (95%CI, 6.49, 8.36, 14% relative increase) than priority municipalities (4.32 (95%CI, 3.65, 4.99); 10% relative increase). This was driven by greater allocation of PMM doctors to non-priority municipalities and higher rates of substitution of non-PMM doctors in priority municipalities. Notably, PMM introduction was associated with reductions in amenable mortality in priority municipalities (− 1.26 (95%CI, − 2.08, − 0.44), but not in non-priority municipalities.

**Table 2:** Subgroup effects of PMM introduction according to priority under allocation criteria and baseline primary care doctor density

	Primary care doctor density	95%CI	Relative change	PMM doctor density	95%CI	Non-PMM doctor density	95%CI	Amenable mortality	95%CI
**Groups of municipalities by allocation criteria**									
Non-Priority	7.43***	6.49,8.36	14.1%	15.73***	15.18,16.28	−8.30***	−9.21,-7.40	−0.61	− 1.20,0.38
Priority	4.32***	3.65,4.99	10.3%	14.63***	14.22,15.04	−10.31***	−11.01,-9.61	− 1.26**	−2.08,-0.44
**Quintiles of baseline primary care doctor density**									
Q1 (lowest)	9.90***	9.08,10.71	40.2%	10.95***	10.48,11.42	−1.05**	−1.78,-0.31	− 1.19**	−2.04,-0.34
Q2	7.51***	6.72,8.30	21.8%	12.60***	12.03,13.17	−5.09***	−5.83,-4.35	−2.23*	− 2.58,-0.09
Q3	5.33***	4.47,6.19	12.1%	14.46***	13.84,15.08	−9.13***	−9.96,-8.29	−1.69*	−3.01,-0.38
Q4	4.77***	3.72,5.82	9.0%	17.12***	16.39,17.85	−12.35***	−13.35,-11.36	0.53	−0.99,2.04
Q5 (Highest)	0.15	−1.64,1.95	0.1%	21.79***	20.83,22.75	−21.63***	−23.29,-19.98	0.55	−1.25,2.34

* *p* < 0.05, ** *p* < 0.01, *** *p* < 0.001; Cluster robust standard errors employed; Municipalities grouped into priority or non-priority by programme allocation criteria and into five quintiles based on primary care doctor density prior to PMM implementation; Allocation priority and quintiles dummies interacted with dummy variable for PMM introduction to obtain effect sizes for each group from two separate regression models (priority and baseline quintiles) for each outcome; Adjusted for Health expenditure (R$) per capita, Private insurance plan coverage (%), Hospital beds per 1000 pop, GDP per capita, Bolsa Familia expenditure per poor person, Illiteracy rate (15 + year), Households with inadequate sanitation (%), Urbanisation rate (%), Income (R$) per capita, Households with no electricity (%), and state-year-quarter and municipal fixed effects; Doctor densities expressed per 100,000 population. Amenable mortality rate per 100,000 population aged under 75 years

The PMM was associated with larger increases in total primary care doctor density in municipalities that had lower densities of primary care doctors at baseline compared to municipalities with higher baseline doctor density (Table 2). However, this overall increase in total doctors was driven by two opposing trends. Municipalities with higher doctor density at baseline experienced larger increases in PMM doctors following PMM implementation, but also increases in substitution of existing doctors. Thus, Q5 (highest baseline doctor density) received the greatest increase in PMM doctors, but there was near complete substitution of non-PMM doctors and no significant change in overall primary care doctor density following PMM introduction. This compared to municipalities in Q1 (lowest) which had lower increases in PMM density, but the lowest rates of substitution resulting in the largest increases in overall primary care doctor density. Notably, there were reductions in mortality associated with PMM introduction in municipalities Q1, Q2, and Q3 (lower baseline doctor density), with no changes in mortality in municipalities in Q4 and Q5 (the highest).

Differential associations of the PMM by PMM doctor nationality were examined. Total primary care doctor density increased the most in municipalities where PMM doctors were mostly foreign (i.e. non-Brazilian) (Table 3). Whilst these areas had higher increases in PMM doctors, and also higher rates of substitution of non-PMM doctors, they were the only areas where there were reductions in amenable mortality associated with PMM introduction (− 1.50 deaths per 100,000 per year (95%CI,-2.32,-0.69).

**Table 3:** Subgroup effects of PMM implementation according to PMM doctor nationality

Percentage of PMM doctors that are Brazilian in participating municipalities	Primary care doctor density	95%CI	PMM doctor density	95%CI	Non-PMM doctor density	95%CI	Amenable mortality	95%CI
< 20%	6.40***	5.70,7.09	16.04***	15.65,16.43	−9.64***	−10.34,-8.95	− 1.50***	−2.32,-0.69
20–80%	5.73***	4.92,6.55	16.99***	16.40,17.58	−11.26***	−12.12,-10.40	−0.92	− 1.84,0.01
> 80%	3.63***	2.67,4.58	10.88***	10.33,11.43	−7.25***	−8.24,-6.26	−0.35	−1.34,0.64

* *p* < 0.05, ** *p* < 0.01, *** *p* < 0.001; Cluster robust standard errors employed; PMM implementation variable divided into three categories based on percentage of PMM doctors that were Brazilian; Dummies denoting categories interacted with dummy variable for PMM implementation to obtain effect sizes for each category in one regression model per each outcome; Adjusted for Health expenditure (R$) per capita, Private insurance plan coverage (%), Hospital beds per 1000 pop, GDP per capita, Bolsa Familia expenditure per poor person, Illiteracy rate (15 + year), Households with inadequate sanitation (%), Urbanisation rate (%), Income (R$) per capita, Households with no electricity (%), and state-year-quarter and municipal fixed effects; Doctor densities expressed per 100,000 population. Amenable mortality rate per 100,000 population aged under 75 years

### Sensitivity analyses

Models were robust to alternative time and state fixed effects specifications (see Additional file 1). Stepwise addition of covariates indicated the stability of the modelling approach. The PROVAB programme only contributed a small proportion of doctors entering the PMM (1544 FTEs in Q4 2015, 9.0% of the total PMM FTEs). Adjusting the PMM analysis to remove these doctors did not alter the main findings and effect sizes. Alternative specifications of covariates including interactions of linear time trends with baseline covariates did not substantially alter our findings. Furthermore, the use of IPTW revealed similar findings with PMM introduction associated with a lower amenable mortality rate of − 1.61 deaths per 100,000 (95%CI, − 2.72 to − 0.49) per year. Event study approaches demonstrated the validity of the analytical approach and confirmed the main findings.

## Discussion

This study found the PMM in Brazil led to increases in primary care doctor density and was associated modest (1.4%) reductions in mortality amenable to healthcare. However, several findings indicate that programme impacts were lower than might be anticipated. The original criteria for prioritising municipalities to receive PMM doctors was not adhered to (81.0% of municipalities received a PMM doctor but only 46.5% met criteria), and there was large-scale substitution of existing primary care doctors. As a result, municipalities prioritised under programme criteria received fewer PMM doctors than non-priority municipalities where substitution effects were greater. Likewise, municipalities with a greater need for doctors received the smallest increases in PMM doctors.

Allocation of doctors to non-priority areas and local substitution effects likely undermined potential health benefits of PMM. Any municipality requesting a PMM doctor received at least one doctor. The substitution of existing doctors, particularly in areas with the greater primary care doctors at baseline, may be explained by a few factors. Although formal substitution was not allowed, more stringent attendance, training and supervision requirements for PMM doctors (particularly the Cubans) may have prompted existing doctors with high rates of absenteeism to leave [34]. Additionally, existing Brazilian doctors may have joined PMM due to higher remuneration and reliable salaries (from federal as opposed to municipal governments). Another factor is that participation in PMM by Brazilian doctors for a minimum of one year conferred an advantage in competition to enter residency in other, “more prestigious” medical specialties.

The finding that expanding supply of primary care doctors is associated with reductions in amenable mortality is concordant with evidence showing mortality reductions (including amenable [10], cardiovascular [12] and infant mortality [35, 36]) following ESF expansion. It is also inline with broader international evidence on health benefits from expanding primary care services [37, 38] and the relationship between HRH and health outcomes [39]. The findings also align with evidence showing reductions in hospitalisations following PMM introduction [20, 21, 28, 29] and support a causal interpretation of population health improvement. Mortality reductions could come from a range of mechanisms. Primary care’s role in prevention and resolving basic health needs is important and evidence shows PMM improved access, satisfaction, service quality, and utilisation [18, 19, 21]. Specifically, reductions in respiratory mortality identified could stem from vaccination and access to antibiotics [40, 41]. Mortality benefits could also accrue through increased referral to hospitals with evidence demonstrating PMM’s role in increasing referrals [21]. However, reductions in mortality were small and there may be reasons why other causes of death were not significantly associated with PMM introduction. A few new doctors across a large area may not have substantially improved healthcare access or quality if there were existing health professionals (including nurses and community health workers) already providing care. Some amenable conditions, such as neoplasms and maternal outcomes may be more amenable to secondary care. Also, wider factors likely constrained primary care effectiveness including weak secondary care and a need to address wider social determinants of health [42].

There are limitations pertinent to this study. Firstly, the analytical approach was ecological, prohibiting individual inference and limiting causal interpretation. However, the approach was robust, has been employed by similar studies [10, 11, 21, 28, 29, 43], exploits the quasi-experimental nature of the programme, and provides stronger evidence over most other observational studies. Sensitivity analyses demonstrate the robustness of the findings to alternative specifications in covariates and time trends. Using municipalities as the unit of analysis may also underestimate programme effects if there is targeting to certain populations in municipalities, and smaller units of analysis such as health units could be appropriate in future studies [44]. Secondly, there is potential for bias from data errors and manipulation, and modelling specifications. All data sources come from administrative sources considered of high quality, and sensitivity analyses point to the robustness of the findings. Thirdly, potential bias can come from comparing PMM and non-PMM receiving municipalities given they may be different in certain respects. Other studies employing the same methods point to the validity of the assumptions underpinning the analysis [21, 29], and descriptive trends show parallel trends at baseline (see Additional file 1). Event study approaches further demonstrate the validity of these assumptions (see Additional file 1). Fourthly, the amenable mortality metric is limited as it also includes conditions sensitive to secondary care and may have limited identification of mortality effects directly related to primary care. Fifthly, the PMM may have prompted better recording and doctor allocation by local health system managers, and the substitution effect could partly have come from absent doctors being removed from the system.

Evidence is growing that large-scale health system interventions to address HRH supply shortages can deliver health gains [1, 20]. However, this study demonstrates the importance of developing and adhering to comprehensive needs-based criteria for HRH allocation. The PMM could have delivered a near 30% increase in primary care doctors (a peak of 18,000 PMM doctors added to the 61,000 primary care doctor workforce in Brazil during 2013), but the actual increase was only 12.2% as nearly two thirds of PMM doctors substituted existing primary care doctors. The programme did not deploy a formal, comprehensive needs-based approach for doctor allocation [22] and widespread allocation of PMM doctors to non-prioritised municipalities in addition to local substitution effects mean full health benefits of PMM were not realised. Improved targeting and implementation of HRH interventions is needed to maximize benefits and to drive progress towards UHC and the SDGs - especially in LMICs.

Political, administrative and financial factors likely undermined the PMM’s effectiveness in Brazil, but a wider question remains around the longer-term sustainability of programmes which import foreign health professionals to address domestic shortages in HRH. This was an issue for the PMM with the withdrawal of all Cuban doctors by the Cuban government in November 2018. The introduction of Cuban doctors was an “emergency” component of the PMM, and there were efforts to primary care clinics and training positions for doctors. Despite funding shortages, these longer-term sustainable solutions to appropriately train and distribute sufficient high-quality professionals domestically must remain a key priority. In December 2019, Brazil introduced the *“Medicos Pelo Brasil”* (Doctors for Brazil) programme to replace the PMM. It aims fill 18,000 positions with Brazilian doctors in underserved areas incentivized with higher salaries for Brazilian doctors and increased training. However, the despite the departure of the Cuban doctors, many positions remain unfilled and the 2020 COVID-19 pandemic in Brazil has significantly disrupted plans to recruit Brazilian doctors, with some Cuban doctors returning to take over empty positions. Great uncertaintly remains over how future policies will tackle Brazil’s sizeable inequalities in HRH. Furthermore, the Brazilian government has introduced deep austerity measures restricting public health expenditures for the next 20 years [45]. This is likely to a have major impact on financing for HRH in the future and, combined with withdrawal of Cuban doctors, negative impacts on population health [46, 47].

## Conclusion

The recent entry of over 35,000 doctors into primary care in Brazil through PMM serves as an internationally important natural experiment in HRH policy. The widespread allocation of PMM doctors to non-priority areas and local substitution effects undermined programme impacts, including contributing to relatively modest reductions in amenable mortality. Policymakers should recognize the importance of developing policies to expand HRH provision in improving health, but must prioritize actions to maximize the benefits and ensure policy effectiveness including developing and implementing comprehensive needs-based criteria.

## Supplementary information

**Additional file 1.** Supplementary material. Appendices and supplementary analyses.

## Data Availability

All data used in this study are available from public sources (see citations in text), but data used that support the findings of this study are available from the corresponding author upon reasonable request.

## Abbreviations

PMM: More Doctors Programme
SDGs: Sustainable Development Goals
HRH: Human Resources for Health
LMICs: Low- and middle-income countries
ESF: Estratégia Saúde da Família
GDP: Gross Domestic Production
FTE: Full time equivalents
