# Supplementary material for: Impact of the Programa Mais médicos (more doctors Programme) on primary care doctor supply and amenable mortality: quasi-experimental study of 5565 Brazilian municipalities
Source: BMC Health Serv Res. 2020 Sep 15;20:873. doi: 10.1186/s12913-020-05716-2 (PMC7491024; doi:10.1186/s12913-020-05716-2)
Supplement: Supplementary file 1 — Additional file 1. Supplementary material. Appendices and supplementary analyses. [file 12913_2020_5716_MOESM1_ESM.docx]

**Supplementary material for: Impact of the Programa Mais Medicos (More Doctors Programme) on primary care doctor supply and amenable mortality: quasi-experimental study of 5565 Brazilian municipalities**

**Table A1 – Amenable mortality International classification of disease codes (ICD-10) and age restrictions**

| **Cause of Death** | **Age** | **ICD-10 Code** | | **Group** |
| --- | --- | --- | --- | --- |
| Intestinal infections | 0-14 | A00-9 | | Infectious diseases |
| Tuberculosis | 0-74 | A15-9, B90 | |  |
| Other infections (diphtheria, tetanus, septicaemia, poliomyelitis) | 0-74 | A36, A35, A40-1, A80 | |  |
| Whooping cough | 0-14 | A37 | |  |
| Measles | 1-11 | B05 | |  |
| Malignant neoplasm of colon and rectum | 0-74 | C18-21 | | Neoplasms |
| Malignant neoplasm of skin | 0-74 | C44 | |  |
| Malignant neoplasm of breast | 0-74 | C50 | |  |
| Malignant neoplasm of cervix uteri | 0-74 | C53 | |  |
| Malignant neoplasm of cervix uteri and body of uterus | 0-44 | C54, C55 | |  |
| Malignant neoplasm of testis | 0-74 | C62 | |  |
| Hodgkin’s disease | 0-74 | C81 | |  |
| Leukaemia | 0-44 | C91-5 | |  |
| Diseases of the thyroid | 0-74 | E00-7 | | Circulatory and endocrine diseases |
| Diabetes | 0-49 | E10-4 | |  |
| Chronic rheumatic heart disease | 0-74 | I05-9 | |  |
| Hypertensive disease | 0-74 | I15 | |  |
| Ischaemic heart disease: 50% of deaths | 0-74 | I20-25 | |  |
| Cerebrovascular disease | 0-74 | I60-9 | |  |
| Congenital cardiovascular anomalies | 0-74 | Q20-8 | |  |
| All respiratory diseases (excl. pneumonia, influenza) | 1-14 | J00-9, J20-99 | | Respiratory disease |
| Influenza | 0-74 | J10-1 | |  |
| Pneumonia | 0-74 | J12-8 | |  |
| Peptic ulcer | 0-74 | K25-7 | | Digestive disorders, genitourinary disease, and epilepsy |
| Appendicitis | 0-74 | K35-8 | |  |
| Abdominal hernia | 0-74 | K40-6 | |  |
| Cholelithiasis and cholecystitis | 0-74 | K80-1 | |  |
| Nephritis and nephrosis | 0-74 | N00-7, N17-9, N25-7 | |  |
| Benign prostatic hyperplasia | 0-74 | N40 | |  |
| Epilepsy | 0-74 | G40-1 | |  |
| Misadventures to patients during surgical and medical care | 0-74 | Y60-9, Y83-4 | | Iatrogenic harm |
| Maternal death | 0-74 | O00-99 | | Maternal and perinatal |
| Perinatal deaths, all causes (excl. stillbirths) | 0-74 | P00-96 | |  |
| Source: Nolte and McKee (2008) |  | |  |  |

**Figure A2 – Number of doctors (FTEs) by sector in Brazil (2008-2017)**

**
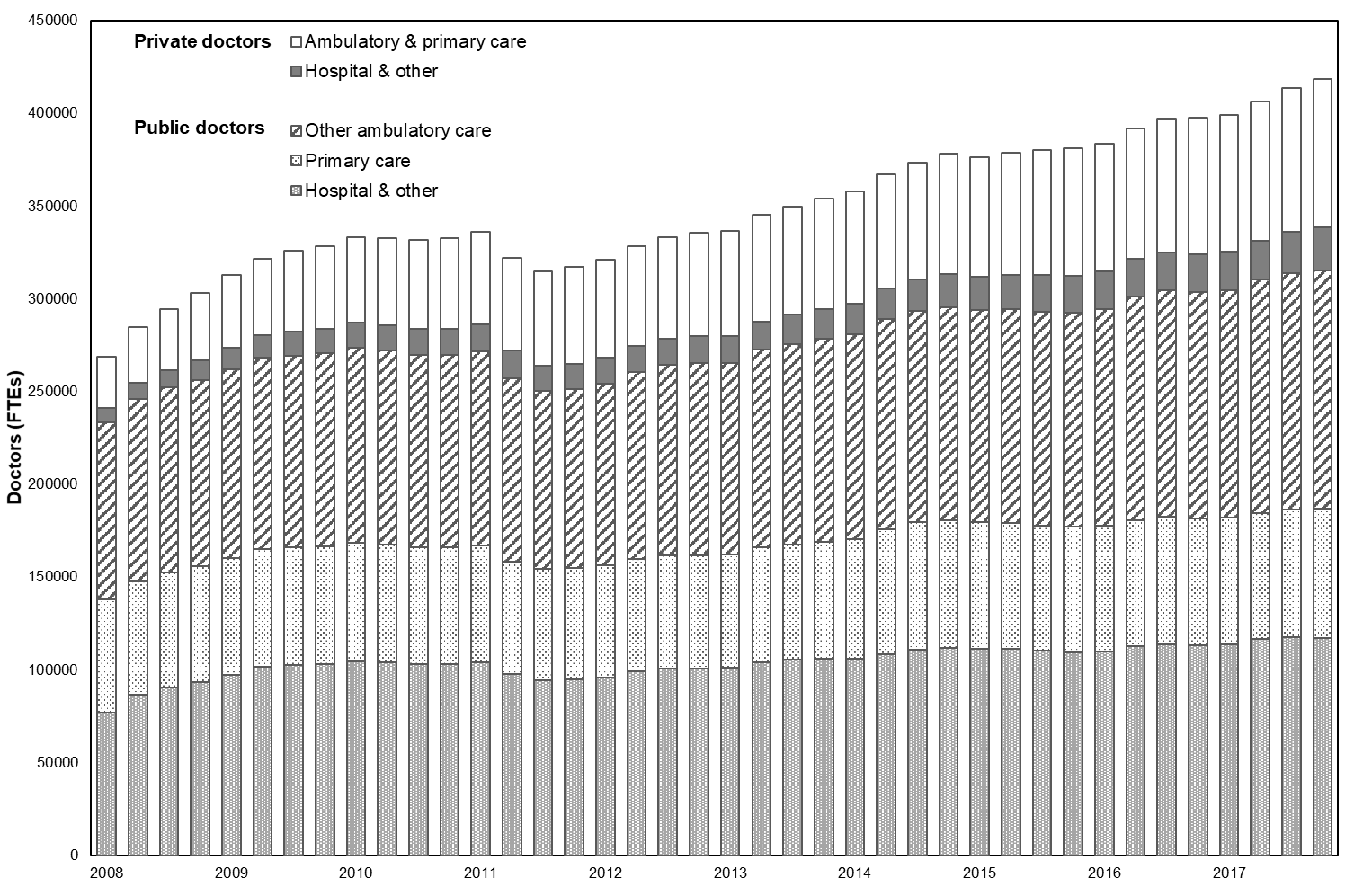
**

Source: CNES, Ministry of Health and author’s own work

**Figure A3 – Number of PMM doctors registered (2013-2017)**

Source: CNES, Ministry of Health and author’s own work

**Figure A4 – Distribution of public doctors in Brazil between hospital care, primary care and other ambulatory care services (2008-2017)**

**
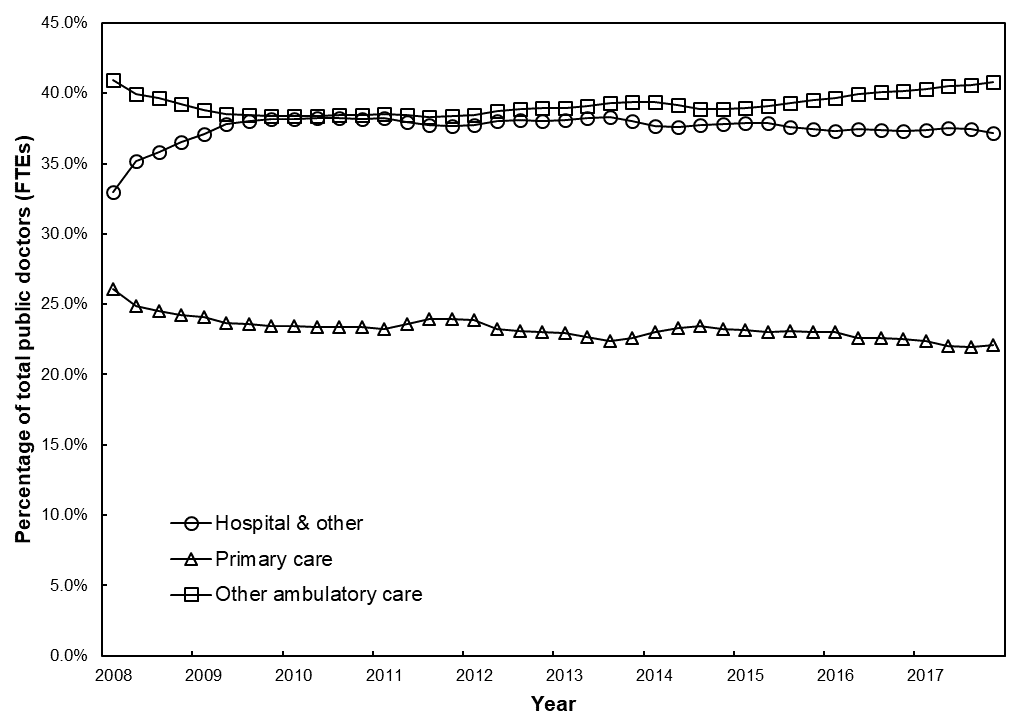
**

Source: CNES, Ministry of Health and author’s own work

**Figure A5 – Number of municipalities with PMM doctors registered (2013-2017)**

**
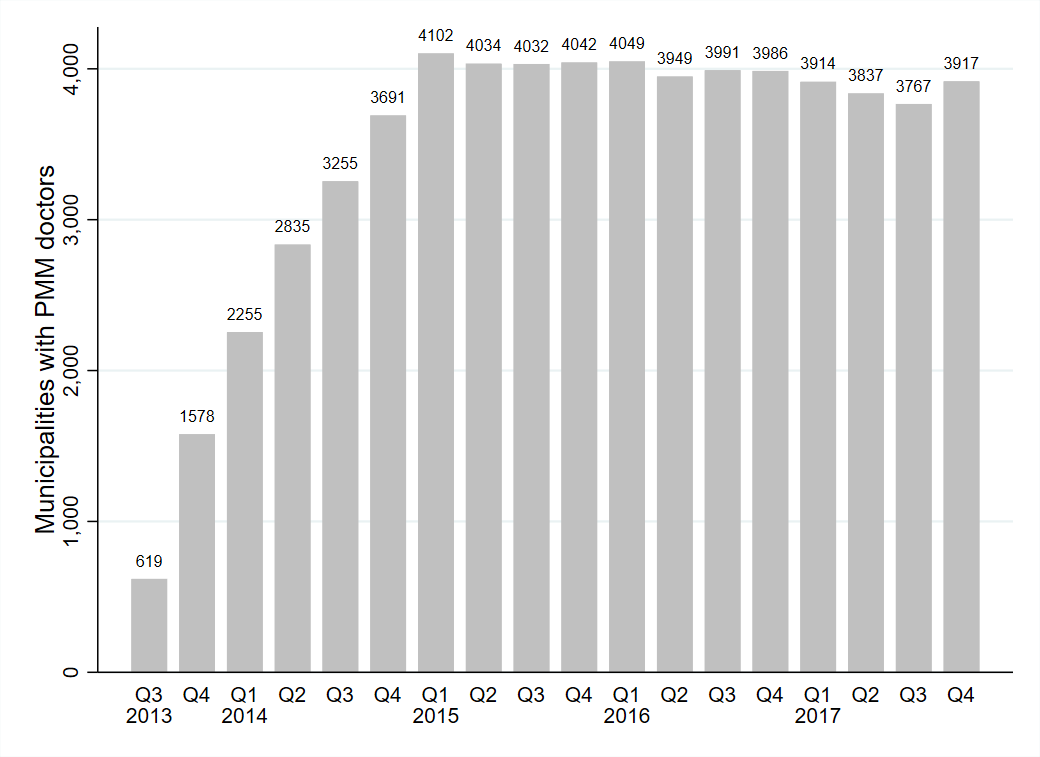
**

Source: CNES, Ministry of Health and author’s own work

**Table A6 – Municipalities receiving PMM by state**

| **State** | **Non-PMM municipalities** | **PMM-municipalities** | **%** |
| --- | --- | --- | --- |
| Rondônia | 0 | 52 | 100% |
| Acre | 1 | 21 | 95% |
| Amazonas | 0 | 62 | 100% |
| Roraima | 0 | 15 | 100% |
| Pará | 3 | 140 | 98% |
| Amapá | 0 | 16 | 100% |
| Tocantins | 51 | 88 | 63% |
| Maranhão | 21 | 196 | 90% |
| Piauí | 58 | 166 | 74% |
| Ceará | 4 | 180 | 98% |
| Rio Grande do Norte | 25 | 142 | 85% |
| Paraíba | 44 | 179 | 80% |
| Pernambuco | 7 | 178 | 96% |
| Alagoas | 15 | 87 | 85% |
| Sergipe | 7 | 68 | 91% |
| Bahia | 18 | 399 | 96% |
| Minas Gerais | 250 | 603 | 71% |
| Espírito Santo | 10 | 68 | 87% |
| Rio de Janeiro | 7 | 85 | 92% |
| São Paulo | 214 | 431 | 67% |
| Paraná | 72 | 327 | 82% |
| Santa Catarina | 58 | 235 | 80% |
| Rio Grande do Sul | 92 | 404 | 81% |
| Mato Grosso do Sul | 11 | 67 | 86% |
| Mato Grosso | 26 | 115 | 82% |
| Goiás | 59 | 187 | 76% |
| Brasilia (Federal District) | 0 | 1 | 100% |

Source: Ministry of Health and author’s own work

**Table A7 – Baseline doctor density and mean PMM doctors by programme priority**

|  | **Mean** | **Min** | **Max** | **Non PMM municipalities** | **PMM municipalities** |
| --- | --- | --- | --- | --- | --- |
| **Baseline doctor density** (Primary care doctors per 100,000 (2008-2012)) | | | | | |
| Non-priority | 52.0 | 5.1 | 197.1 | 1,056 | 1,920 |
| Priority | 41.6 | 0.9 | 150.3 | 0 | 2,589 |
|  |  |  |  |  |  |
| **Mean PMM doctor density** (PMM doctors per 100,000 (2013-2017)) | | | | | |
| Non-priority | 16.2 | 0.2 | 72.4 | 1,056 | 1,920 |
| Priority | 15.4 | 0.1 | 66.0 | 0 | 2,589 |

Source: CNES, Ministry of Health and author’s own work

**Table A8 – Baseline doctor density and mean PMM doctors by quintiles of municipalities**

|  | **Mean** | **Min** | **Max** | **Non PMM municipalities** | **PMM municipalities** |
| --- | --- | --- | --- | --- | --- |
| **Baseline doctor density** (Primary care doctors per 100,000 (2008-2012)) | | | | | |
| Q1 (lowest) | 23.6 | 0.9 | 30.6 | 89 | 1024 |
| Q2 | 35.4 | 30.6 | 39.5 | 149 | 964 |
| Q3 | 43.4 | 39.5 | 47.5 | 209 | 904 |
| Q4 | 52.9 | 47.5 | 60.0 | 240 | 873 |
| Q5 (highest) | 80.6 | 60.0 | 197.1 | 369 | 744 |
|  |  |  |  |  |  |
| **Mean PMM doctor density** (PMM doctors per 100,000 (2013-2017)) | | | | | |
| No PMM | 0 | 0 | 0 | 1056 | 0 |
| Q1 (lowest) | 3.9 | 0.1 | 6.5 | 0 | 902 |
| Q2 | 8.8 | 6.6 | 11.1 | 0 | 902 |
| Q3 | 13.5 | 11.1 | 16.1 | 0 | 902 |
| Q4 | 19.6 | 16.1 | 23.4 | 0 | 902 |
| Q5 (highest) | 32.8 | 23.4 | 72.4 | 0 | 901 |

Source: CNES, Ministry of Health and author’s own work

**Figure A9 – Numbers of municipalities by month of receiving first MM doctor**

Source: CNES, Ministry of Health and author’s own work.

**Appendix A10 – Estimation strategy**

The equation used to estimate this

*y_mq_ = β_1_X_1_ + β_n_X_n_ + a_m_ + q + T_s_ + ε*

Where:
*y_mq_* is the outcome (e.g. amenable mortality rate) in municipality *m* in year-quarter *q*;

*β_1_* is the coefficient for binary variable *X_1_* (the introduction of the PMM) in a municipality in municipality *m* in year-quarter *q* ;

*β_n_* are the coefficients for a range of covariates *X_n_* ;

*a_m_* is the municipal fixed effect;

*q* is the year-quarter fixed effect (e.g. 2010 Q1; 2010 Q2; 2012 Q3 etc);

*T_s_* are linear time trends for each State *s*; and

*ε* is the unestimated error term;

**A11 Table - Univariate panel regression analyses on amenable mortality**

The table below shows univariate regression analysis for each covariate. Each line represents a different regression model. Each model controls for municipality and year-quarter fixed effects and linear state time trends.

|  | **Amenable Mortality Rate** | **95% CI** |
| --- | --- | --- |
| PMM Implementation | -1.244*** | -1.968,-0.520 |
| Health expenditure (R$) per capita | 2.272 | -0.928,5.472 |
| Private insurance plan coverage (%) | 0.356*** | 0.276,0.432 |
| Hospital beds per 1000 pop | 0.872*** | 0.456,1.288 |
| GDP per capita | -1.120 | -33.196,30.956 |
| Bolsa Familia expenditure per poor person | 0.008* | 0.001,0.016 |
| Illiteracy rate (15+year) | 0.164 | -0.192,0.516 |
| Households with inadequate sanitation (%) | -0.160** | -0.280,-0.040 |
| Urbanisation rate (%) | 0.116* | 0.012,0.216 |
| Income (R$) per capita | -0.016*** | -0.028,-0.008 |
| Households with no electricity (%) | 0.200* | 0.040,0.360 |

* p<0.05, ** p<0.01, *** p<0.001; Amenable mortality rate is expressed per 100,000 population and reported as annual effect sizes; Heteroskedastic and Autocorrelation cluster-robust standard errors employed;

**A12 Table – Sequential addition of covariates on panel regression models on amenable mortality**

|  | **β**  **95%CI** | **β**  **95%CI** | **β**  **95%CI** | **β**  **95%CI** | **β**  **95%CI** | **β**  **95%CI** | **β**  **95%CI** | **β**  **95%CI** | **β**  **95%CI** | **β**  **95%CI** | **β**  **95%CI** |
| --- | --- | --- | --- | --- | --- | --- | --- | --- | --- | --- | --- |
| PMM Implementation | -1.244*** | -1.216*** | -1.284*** | -1.304*** | -1.296*** | -1.180*** | -1.284*** | -1.260*** | -1.228*** | -1.096** | -1.056** |
|  | -1.968,-0.520 | -1.960,-0.476 | -2.024,-0.548 | -2.040,-0.564 | -2.036,-0.556 | -1.912,-0.444 | -2.016,-0.548 | -1.996,-0.528 | -1.960,-0.500 | -1.820,-0.372 | -1.780,-0.332 |
| Health expenditure (R$) per capita | - | 2.184 | 1.736 | 1.632 | 1.992 | 2.348 | 2.164 | 2.196 | 2.152 | 3.008* | 2.956* |
|  |  | -0.964,5.336 | -1.164,4.640 | -1.236,4.504 | -1.012,5.000 | -0.676,5.372 | -0.868,5.192 | -0.802,5.216 | -0.832,5.140 | 0.088,5.924 | 0.052,5.856 |
| Private insurance plan coverage (%) | - | - | 0.352*** | 0.348*** | 0.352*** | 0.376*** | 0.376*** | 0.376*** | 0.376*** | 0.352*** | 0.336*** |
|  |  |  | 0.272,0.432 | 0.268,0.428 | 0.272,0.428 | 0.296,0.456 | 0.292,0.456 | 0.296,0.460 | 0.296,0.456 | 0.276,0.428 | 0.26,0.412 |
| Hospital beds per 1000 pop | - | - | - | 0.804*** | 0.808*** | 0.776*** | 0.772*** | 0.780*** | 0.780*** | 0.804*** | 0.808*** |
|  |  |  |  | 0.400,1.212 | 0.404,1.216 | 0.368,1.18 | 0.368,1.18 | 0.376,1.184 | 0.376,1.188 | 0.404,1.208 | 0.404,1.208 |
| GDP per capita | - | - | - | - | -20.788 | -14.588 | -17.240 | -16.656 | -16.508 | -7.848 | -7.808 |
|  |  |  |  |  | -48.480,6.928 | -42.760,13.588 | -45.280,10.816 | -44.760,11.448 | -44.560,11.548 | -34.784,19.084 | -34.680,19.060 |
| Bolsa Familia expenditure per poor person | - | - | - | - | - | 0.012*** | 0.016*** | 0.016*** | 0.016*** | 0.016*** | 0.020*** |
|  |  |  |  |  |  | 0.004,0.02 | 0.008,0.024 | 0.008,0.028 | 0.008,0.024 | 0.008,0.024 | 0.008,0.028 |
| Illiteracy rate (15+year) | - | - | - | - | - | - | 0.448* | 0.440* | 0.492** | 0.784*** | 0.628*** |
|  |  |  |  |  |  |  | 0.092,0.800 | 0.080,0.796 | 0.136,0.848 | 0.428,1.140 | 0.284,0.972 |
| Households with inadequate sanitation (%) | - | - | - | - | - | - | - | -0.180** | -0.176** | -0.152* | -0.136* |
|  |  |  |  |  |  |  |  | -0.296,-0.064 | -0.292,-0.060 | -0.272,-0.036 | -0.252,-0.020 |
| Urbanisation rate (%) | - | - | - | - | - | - | - | - | 0.096* | 0.092* | 0.104* |
|  |  |  |  |  |  |  |  |  | 0.008,0.188 | 0.004,0.184 | 0.016,0.192 |
| Income (R$) per capita | - | - | - | - | - | - | - | - | - | -0.020*** | -0.020*** |
|  |  |  |  |  |  |  |  |  |  | -0.028,-0.008 | -0.028,-0.012 |
| Households with no electricity (%) | - | - | - | - | - | - | - | - | - | - | 0.288*** |
|  |  |  |  |  |  |  |  |  |  |  | 0.120,0.456 |

* p<0.05, ** p<0.01, *** p<0.001; Heteroskedastic and Autocorrelation cluster-robust standard errors employed; Amenable mortality rate is expressed per 100,000 population and reported as annual effect sizes; All models adjusted for municipality and year-quarter fixed effects and linear state time trends; Regression results are weighted by municipal population.

**A13 Table – Panel regression on amenable mortality with linear-trend covariate specification**

The table below shows the results from a fixed effects panel regression on amenable mortality where covariates are specified as baseline values (Q1 2008) interacted with linear time trends.

|  | **Amenable Mortality Rate** | **95% CI** |
| --- | --- | --- |
| PMM Implementation | -0.756* | -1.484,-0.028 |
|  |  |  |
| **Linear time trends interacted with:** |  |  |
| Health expenditure (R$) per capita | 0.016 | -0.188,0.216 |
| Private insurance plan coverage (%) | -0.004* | -0.004,0.000 |
| Hospital beds per 1000 pop | -0.016** | -0.024,-0.004 |
| GDP per capita | -0.628 | -3.664,2.404 |
| Bolsa Familia expenditure per poor person | 0.000 | 0.000,0.000 |
| Illiteracy rate (15+year) | -0.004 | -0.012,0.004 |
| Households with inadequate sanitation (%) | -0.004 | -0.004,0.000 |
| Urbanisation rate (%) | 0.000 | 0.000,0.000 |
| Income (R$) per capita | 0.000* | 0.000,0.000 |
| Households with no electricity (%) | -0.004 | -0.008,0.000 |
|  |  |  |
| N (municipalities) | 5565 |  |
| N (observations) | 222600 |  |

* p<0.05, ** p<0.01, *** p<0.001; Amenable mortality rate is expressed per 100,000 population and reported as annual effect sizes; Heteroskedastic and Autocorrelation cluster-robust standard errors employed;

**Figure A14 - Mean amenable mortality rates of MM-receiving and non-MM receiving municipalities (2008-2017)**

Mean rates weighted by municipal population;

**Table A15 – Results from longitudinal fixed effects regression models on groups of amenable mortality**

| **Amenable mortality group** | **PMM introduction** | **95%CI** |
| --- | --- | --- |
| Infectious diseases | 0.024 | -0.036,0.084 |
| Neoplasms | -0.067 | -0.138,0.003 |
| Circulatory and endocrine diseases | -0.050 | -0.141,0.040 |
| Respiratory disease | -0.167*** | -0.250,-0.084 |
| Digestive disorders, genitourinary disease, and epilepsy | -0.011 | -0.067,0.044 |
| Iatrogenic harm | 0.011 | -0.001,0.024 |
| Maternal and perinatal | -0.009 | -0.081,0.064 |

* p<0.05, ** p<0.01, *** p<0.001; Heteroskedastic and Autocorrelation cluster-robust standard errors employed; regression models weighted by municipal population size; Each effect size for PMM introduction from a separate regression model adjusted for: health expenditure (R$) per capita, private insurance plan coverage (%), hospital beds per 1000 pop, GDP per capita, Bolsa Familia expenditure per poor person, illiteracy rate (15+year), households with inadequate sanitation (%), urbanisation rate (%), income (R$) per capita, households with no electricity (%), and state-year-quarter and municipal fixed effects.

**Table A16 – Sensitivity of longitudinal regression models on amenable mortality to varying time and fixed effects specifications**

|  | **Model 1** | **95%CI** | **Model 2** | **95%CI** | **Model 3** |  |
| --- | --- | --- | --- | --- | --- | --- |
| PMM introduction | -1.264** | -2.024,-0.504 | -1.220** | -2.004,-0.440 | -1.300*** | -2.052,0.552 |
|  |  |  |  |  |  |  |
| Municipality fixed effects | Y |  | Y |  | Y |  |
| Quarter-year fixed effects | Y |  | - |  | Y |  |
| State-quarter-year fixed effects | - |  | Y |  | - |  |
| State year-quarter linear trends | - |  | - |  | Y |  |
|  |  |  |  |  |  |  |
| N (Municipalities) | 5565 |  | 5565 |  | 5565 |  |
| N (Observations) | 222600 |  | 222600 |  | 222600 |  |
| AIC | 1523009 |  | 1521516 |  | 1522574 |  |
| BIC | 1523421 |  | 1531293 |  | 1523244 |  |

* p<0.05, ** p<0.01, *** p<0.001; Heteroskedastic and Autocorrelation cluster-robust standard errors employed; regression models weighted by municipal population size; Results from longitudinal regression models on amenable mortality.

**Table A17 – Longitudinal regression models on amenable mortality including slope and step changes following PMM introduction**

|  | **Model 1 – step change**  **(same as main analysis)** | | **Model 2 – step and slope change** | |
| --- | --- | --- | --- | --- |
|  | **β** | **95%CI** | **β** | **95%CI** |
| Step change | -1.060** | -1.784,-0.336 | -0.800* | -1.572,-0.032 |
| Slope change (quarters since PMM introduction) | - |  | 0.100 | -0.096,0.296 |
|  |  |  |  |  |
| N | 222600 |  | 180360 |  |

* p<0.05, ** p<0.01, *** p<0.001; Cluster robust standard errors employed; Adjusted for Health expenditure (R$) per capita, Private insurance plan coverage (%), Hospital beds per 1000 pop, GDP per capita, Bolsa Familia expenditure per poor person, Illiteracy rate (15+year), Households with inadequate sanitation (%), Urbanisation rate (%), Income (R$) per capita, Households with no electricity (%), and state-year-quarter and municipal fixed effects; Doctor densities expressed per 100,000 population. Amenable mortality rate per 100,000 population aged under 75 years.

**Table A18 – Sensitivity analysis using inverse probability weighting of treatment with regression adjustment (IPTW-RA)**

Logistic regression models were carried out on the likelihood of a municipality ever receiving a PMM doctor (binary outcome) to calculate predicted probabilities of PMM introduction use and generate inverse probability weighting of treatment (IPTW). The predictive model was a cross-sectional analysis of municipalities in 2010 and adjusted for municipal primary care coverage, Gini index, index of human development, percentage of households without electricity, mean household income, percentage of households with inadequate sanitation, municipal illiteracy rate of those age 15 years or more, municipal expenditure on Bolsa Familia, private health insurance coverage, hospital beds per 100,000, health expenditure per capita, and public primary care doctors per 100,000.

The IPTW was estimated from this predicted probability using the equation:

$$IPTW= \frac{Z}{e}+\frac{1-Z}{1-e}$$

Where *Z* refers to a binary variable denoting actual treatment (PMM ever introduced; either 0 or 1), and *e* refers to the predicted probability of receiving PMM from the logistic regression model.

Using IPTW, the main regression models were repeated.

|  | **Main regression results (population weighted only)** | | **IPTW regression results (IPTW and population weighted)** | |
| --- | --- | --- | --- | --- |
|  | **Amenable mortality rate** | **95%CI** | **Amenable mortality rate** | **95%CI** |
| PMM Introduction | -1.06** | -1.78,-0.33 | -1.608** | -2.724,-0.492 |
| Health expenditure (R$) per capita | 2.95* | 0.17,5.86 | 2.932 | -0.204,6.068 |
| Private insurance plan coverage (%) | 0.34*** | 0.26,0.41 | 0.292*** | 0.188,0.396 |
| Hospital beds per 1000 pop | 0.81*** | 0.40,1.21 | 0.536* | 0.004,1.068 |
| GDP per capita | -7.81 | -34.68,19.06 | 0.192 | -33.516,33.900 |
| Bolsa Familia expenditure per poor person | 0.02*** | 0.02,0.03 | 0.016** | 0.004,0.024 |
| Illiteracy rate (15+year) | 0.63*** | 0.28,0.97 | 0.816*** | 0.392,1.240 |
| Households with inadequate sanitation (%) | -0.14* | -0.25,-0.02 | -0.064 | -0.204,0.076 |
| Urbanisation rate (%) | 0.10* | 0.02,0.29 | 0.168** | 0.048,0.292 |
| Income (R$) per capita | -0.02*** | -0.03,-0.01 | -0.028*** | -0.040,-0.016 |
| Households with no electricity (%) | 0.29*** | 0.12,0.46 | 0.248* | 0.004,0.456 |
|  |  |  |  |  |
| N (municipalities) | 5565 |  | 5565 |  |
| N (observations) | 222600 |  | 222600 |  |
|  |  |  |  |  |

* p<0.05, ** p<0.01, *** p<0.001; Heteroskedastic and Autocorrelation cluster-robust standard errors employed;

**References**

Austin PC, Stuart EA. Moving towards best practice when using inverse probability of treatment weighting (IPTW) using the propensity score to estimate causal treatment effects in observational studies. Statistics in medicine. 2015;34(28):3661-79.

**Appendix A19 - Event study analyses with leads and lags dummies for PMM introduction on amenable mortality and doctor density**

Primary care doctor density


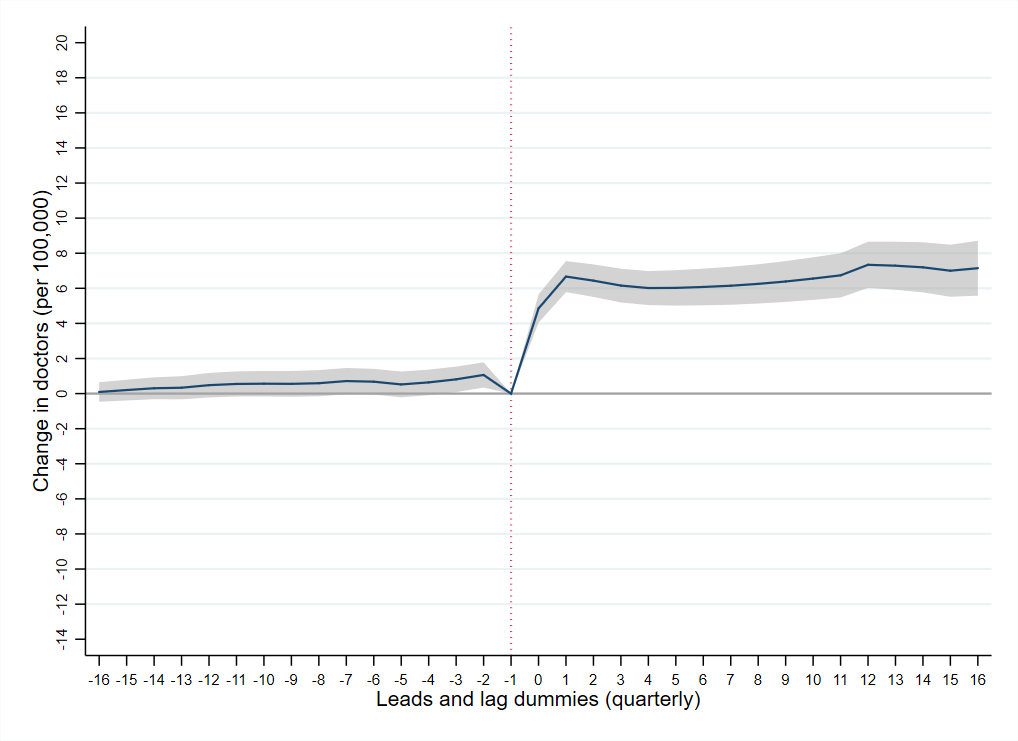


Non-PMM doctor density


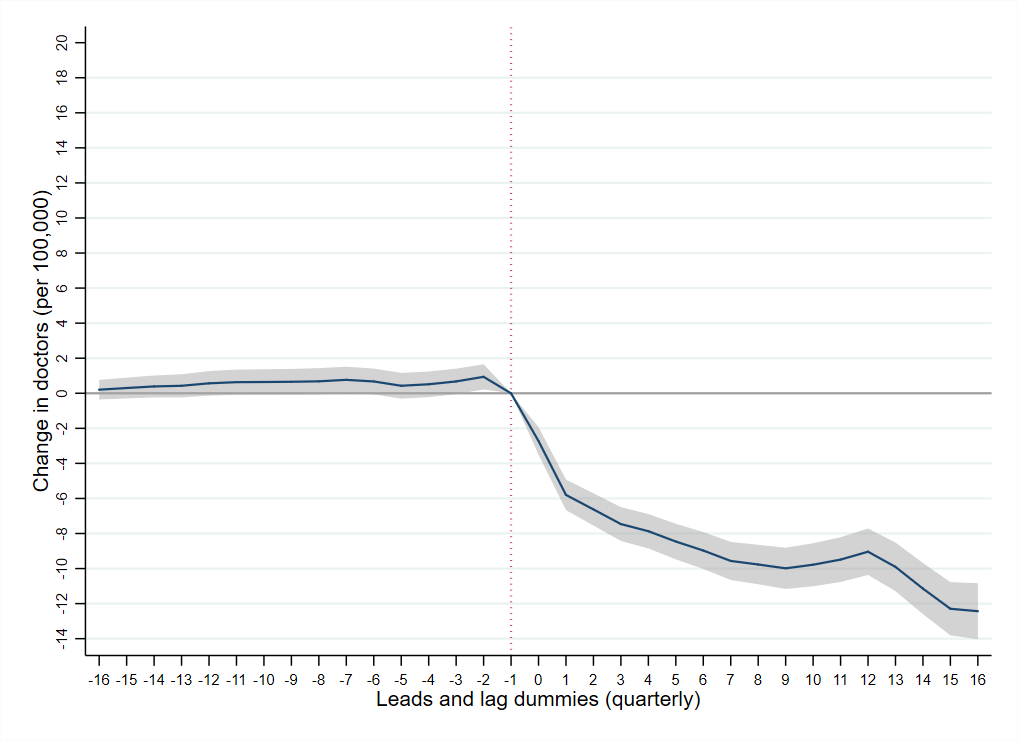


PMM doctor density


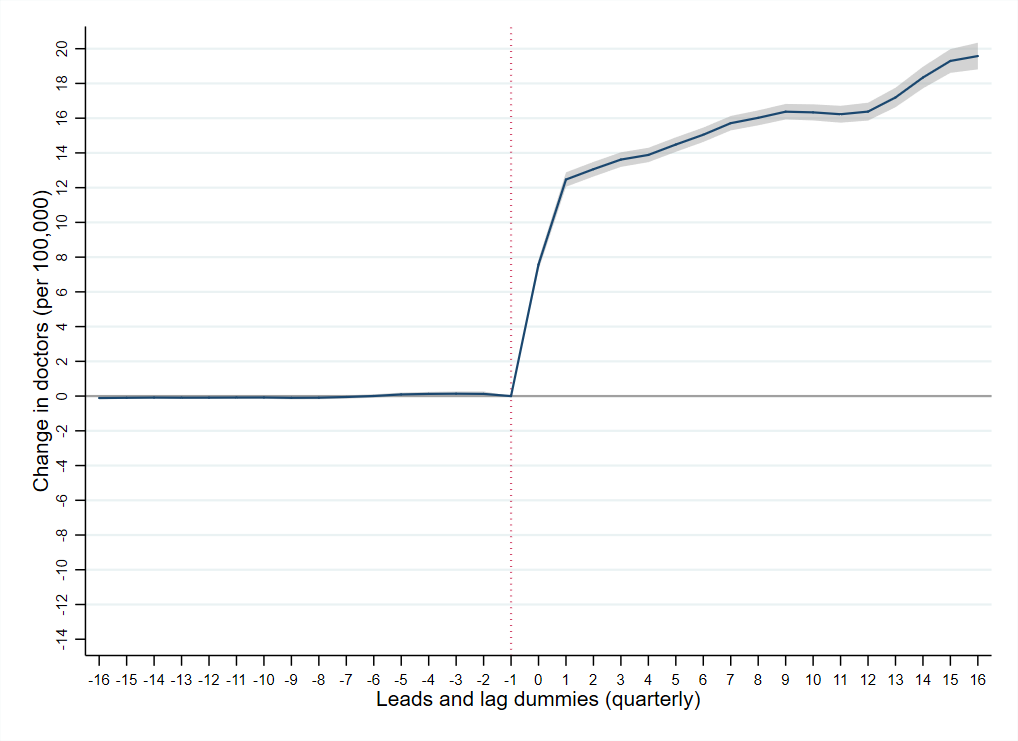


Heteroskedastic and autocorrelation cluster-robust standard errors employed; lead and lag dummies refer to quarter-year periods before and after PMM introduction in a municipality.

Amenable mortality

**
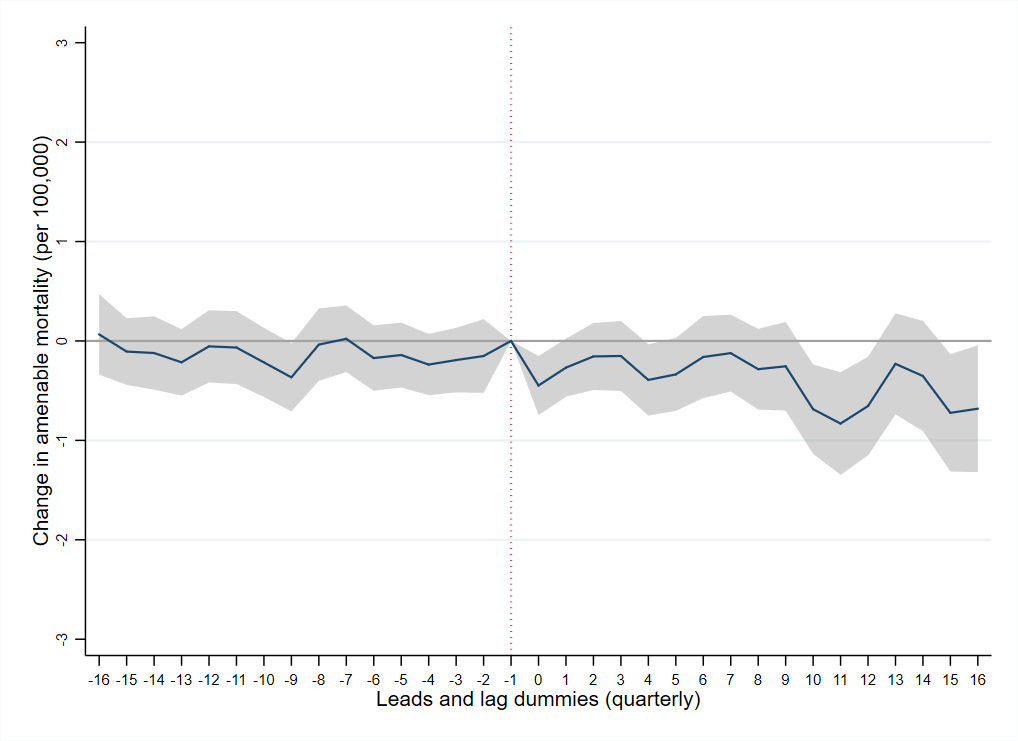
**

Heteroskedastic and autocorrelation cluster-robust standard errors employed; regression model weighted by municipal population size; lead and lag dummies refer to quarter-year periods before and after PMM introduction in a municipality.
